# Prevalence and Risk Factors for School-Associated Transmission of SARS-CoV-2

**DOI:** 10.1001/jamahealthforum.2023.2310

**Published:** 2023-08-04

**Authors:** Sandra B. Nelson, Caitlin M. Dugdale, Isaac Ravi Brenner, Allison Crawford, Alyssa Bilinski, Duru Cosar, Nira R. Pollock, Andrea Ciaranello

**Affiliations:** 1 Division of Infectious Diseases, Massachusetts General Hospital, Boston; 2 Harvard Medical School, Boston, Massachusetts; 3 Medical Practice Evaluation Center, Massachusetts General Hospital, Boston; 4 Department of Health Services, Policy and Practice and Department of Biostatistics, Brown School of Public Health, Providence, Rhode Island; 5 Department of Laboratory Medicine, Boston Children’s Hospital, Boston, Massachusetts

## Abstract

**Importance:**

School-associated SARS-CoV-2 transmission is described as uncommon, although the true transmission rate is unknown.

**Objective:**

To identify the SARS-CoV-2 secondary attack rate (SAR) in schools and factors associated with transmission.

**Design, Setting, and Participants:**

This cohort study examined the risk of school-based transmission of SARS-CoV-2 among kindergarten through grade 12 students and staff in 10 Massachusetts school districts during 2 periods: fall 2020/spring 2021 (F20/S21) and fall 2021 (F21). School staff collected data on SARS-CoV-2 index cases and school-based contacts, and SAR was defined as the proportion of contacts acquiring SARS-CoV-2 infection.

**Exposure:**

SARS-CoV-2.

**Main Outcomes and Measures:**

Potential factors associated with transmission, including grade level, masking, exposure location, vaccination history, and Social Vulnerability Index (SVI), were analyzed using univariable and multivariable logistic regression models.

**Results:**

For F20/S21, 8 school districts (70 schools, >33 000 students) were included and reported 435 index cases (151 staff, 216 students, and 68 missing role) with 1771 school-based contacts (278 staff, 1492 students, and 1 missing role). For F21, 5 districts (34 schools, >18 000 students) participated and reported 309 index cases (37 staff, 207 students, and 65 missing role) with 1673 school-based contacts (107 staff and 1566 students). The F20/S21 SAR was 2.2% (lower bound, 1.6%; upper bound, 26.7%), and the F21 SAR was 2.8% (lower bound, 2.6%; upper bound, 7.4%). In multivariable analysis, during F20/S21, masking was associated with a lower odds of transmission compared with not masking (odds radio [OR], 0.12; 95% CI, 0.04-0.40; *P* < .001). In F21, classroom exposure vs out-of-classroom exposure was associated with increased odds of transmission (OR, 2.47; 95% CI, 1.07-5.66; *P* = .02); a fully vaccinated vs unvaccinated contact was associated with a lower odds of transmission (OR, 0.04; 95% CI, 0.00-0.62; *P* < .001). In both periods, a higher SVI was associated with a greater odds of transmission.

**Conclusions and Relevance:**

In this study of Massachusetts schools, the SAR for SARS-CoV-2 among school-based contacts was low during 2 periods, and factors associated with transmission risk varied over time. These findings suggest that ongoing surveillance efforts may be essential to ensure that both targeted resources and mitigation practices remain optimal and relevant for disease prevention.

## Key Points

**Question:**

What is the rate of secondary transmission of SARS-CoV-2 in schools, and what factors are associated with transmission?

**Findings:**

In this cohort study of 10 Massachusetts school districts, the secondary attack rate of SARS-CoV-2 in schools was 2.2% during the 2020-2021 school year and 2.8% in the fall of 2021. Factors associated with transmission in schools changed over time, although a greater social vulnerability index was associated with transmission in both periods.

**Meaning:**

These findings suggest that although transmission of SARS-CoV-2 in schools was uncommon, ongoing surveillance efforts may be essential to ensure that both targeted resources and mitigation practices remain optimal and relevant for disease prevention.

## Teaser

This cohort study identifies the prevalence of and factors associated with school-based transmission of SARS-CoV-2 infection in Massachusetts public school districts.

## Introduction

When the COVID-19 pandemic first emerged, schools in all 50 states closed as a means of preventing transmission. Over the subsequent 2 years, schools reopened with a variety of mitigation measures to reduce school-associated transmission, including remote and hybrid models to reduce in-school density, distancing requirements, mask mandates, initiation of testing programs, enhanced hand hygiene measures, isolation of symptomatic persons, ventilation improvements, and recommendations for vaccination of students, faculty, and staff. As schools reopened, school-associated transmissions were reported to be uncommon.^1,2,3,4,5^

The true rate of in-school SARS-CoV-2 transmission, however, remains unknown. The impact of mitigation measures in preventing in-school transmission is largely understood at the policy level rather than the individual level, for example, from comparisons between districts with different masking and distancing policies or with different approaches to ventilation.^6,7,8^ Without detailed contact tracing information, factors associated with in-school transmissions are difficult to untangle from transmissions occurring in the community, such as at play dates, during recreational athletic activities, or at after-school gatherings. Furthermore, most of the currently available data on school-associated transmissions were gathered during times when viral variants with lower intrinsic transmissibility than current variants were circulating and prior to the widespread adoption of vaccines.^1^ Understanding how transmission dynamics differ over time and in association with different SARS-CoV-2 prevention measures may inform future strategies around mitigation measures in schools. As schools adapt to this new era in the SARS-CoV-2 pandemic during which waves of disease may continue to occur, data-driven best practices are needed to maximize in-person learning while minimizing transmission risk to students, faculty, and staff. To address this need, we used detailed school-based contact tracing data in a sample of Massachusetts school districts to describe the secondary attack rate (SAR) of SARS-CoV-2 during the 2020-2021 school year and during the fall term of the 2021-2022 school year and identify factors associated with school-based transmissions.

## Methods

### Design

In this cohort study, a convenience sample of 25 Massachusetts public kindergarten through grade 12 (K-12) school districts were invited to participate, and the Massachusetts Department of Elementary and Secondary Education invited all Massachusetts districts to participate in the study through frequent COVID-19 webinars for schools. Interested school districts were provided with a standardized contact tracing spreadsheet (eTable 1 in Supplement 1; eTable 2 in Supplement 2) for the reporting of deidentified data. Data were collected during 2 periods: fall and spring semesters of the 2020-2021 school year (F20/S21), and the fall semester (August 30 through December 8) of the 2021-2022 school year (F21). Districts were encouraged to participate in 1 or both periods if feasible. Ultimately, 8 public school districts contributed F20/S21 data; 3 of these districts plus 1 additional district and 1 private prekindergarten through grade 9 school contributed F21 data. The study was approved by the Mass General Brigham and Massachusetts Department of Public Health institutional review boards. Waiver of informed consent was granted as the data were collected by school personnel as part of their individualized contact tracing programs; only deidentified data were sent to investigators. The study followed the Strengthening the Reporting of Observational Studies in Epidemiology (STROBE) reporting guideline.

### Study Population

Index cases were included if the student, faculty member, or staff member with SARS-CoV-2 was in school while infectious, beginning 48 hours before symptom onset (or collection time of a positive test result if asymptomatic). Requested information about index cases included their role in school (ie, student or staff and grade level or staff role), the means of case identification (eg, regularly scheduled asymptomatic testing, symptomatic testing, testing after exposure in school, testing after exposure outside of school), duration of time spent in school while infectious, and number of in-school close contacts. Specific demographic data (including age, sex, and race and ethnicity) were not collected by school districts as part of their contact tracing efforts and, therefore, could not be included in the analysis. All school-based close contacts were included. Requested information about contacts included role in school (ie, student or staff and grade level or staff role), location of exposure (eg, classroom, lunch or snack time, recess, physical education, bus, school sports, other school-sponsored extracurricular event), and individual mask use during the exposure, as well as whether each contact was tested for SARS-CoV-2 within 14 days following the exposure, the type of test performed, and the test results. The contact tracing tool was updated for F21 to include information about vaccination status, approximate distance between index case and contact at the time of exposure, and the quarantine and testing approach.

Contacts were defined by Massachusetts Department of Public Health criteria^9^ as individuals within 6 feet of an index case for at least 15 minutes (cumulative) over 24 hours during the window of infectiousness. Beginning in April 2021, close contacts within classrooms and on school buses were excluded from quarantine requirements if both the case and contact were masked, unless closer than 3 feet for at least 15 minutes over 24 hours during the window of infectiousness.^10^ During both periods, all Massachusetts school districts were encouraged to participate in weekly asymptomatic screening programs using pooled polymerase chain reaction testing. Seven of the 8 districts offered pooled testing in F20/S21 and 4 of 5 districts offered pooled testing in F21.^10^ For F21, districts were also encouraged to offer a test-to-stay (TTS) program, allowing unvaccinated students and faculty exposed to COVID-19 in schools the option to remain in school with a negative result on daily rapid antigen testing performed by school personnel. All 5 districts offered a TTS program.^11^ Participation in all testing programs was voluntary at the individual level. Vaccination became available in Massachusetts for K-12 staff on March 11, 2021; for students aged 16 years or older on April 19, 2021; for students aged 12 years or older on May 12, 2021; and for students aged 5 to 11 years on November 3, 2021. Vaccination rates among school-aged children varied substantially among participating districts (eFigure in Supplement 3). During F20/S21, fully vaccinated individuals were excluded from contact tracing, according to department of public health guidance. In F20/S21, schools were advised to maintain 3 feet of distance separation in classrooms; many districts operated in a hybrid format, with some students learning remotely. In April 2021, districts were required to offer in-person learning to all students, with no specific requirements regarding distancing. All students were required to return to full in-person learning in F21. Masking was required in classrooms during both periods. During F20/S21, the original SARS-CoV-2 strain was predominant; in F21, the Delta variant was predominant in Massachusetts.

For contacts with positive test results for SARS-CoV-2, the likelihood that transmission occurred in the school setting was assessed by the school-based team. School-based nursing and contact tracing teams designated contacts who tested positive as not a school-associated transmission if a clear alternative exposure was present and believed to be more likely than the school-based exposure (ie, a household contact with exposure timing more convincing for likely source of infection). Transmissions were considered possible school-associated transmissions if there were both school-associated and out-of-school exposures, either of which may have led to transmission. Transmissions were considered probable school-associated transmissions if no out-of-school exposures were identified.

### Statistical Analysis

We defined the SAR as the proportion of school-based contacts acquiring SARS-CoV-2 infection and designated as either possible or probable school-associated transmission. We calculated the SAR in 3 ways: (1) as ascertained by testing, (2) a lower bound (assuming all untested contacts were uninfected), and (3) an upper bound (assuming all untested contacts were infected). We also repeated the SAR calculation in a sensitivity analysis in which all contacts with positive test results (including those deemed not school-associated transmissions) were included. Index cases with 0 school-associated contacts during the window of infectiousness were excluded from analysis. Descriptive analyses were performed to calculate the total number of cases, contacts, and possible or probable school-associated transmission events for each district and in each category of index case and exposure type (student or staff, grade level, exposure setting, masking, etc). In addition, the mean and median number of contacts per case and the proportion of contacts who underwent SARS-CoV-2 testing within 14 days of their exposure were calculated.

We used Fisher exact tests to compare SARs in univariable analysis, with a 2-sided *P* < .05 indicating statistical significance. Unknown or missing values were removed from the univariable analysis if the number of contacts with unknown or missing data comprised less than 5% of the total number of contacts tested for each exposure category; individuals with some missing data could still contribute data in other exposure categories where data were complete. We repeated this calculation twice, assessing the F20/S21 and F21 periods separately due to differences in circulating variants, quarantine and testing policies, and vaccination prevalence. We then fit logistic multivariable regression models for each period, selecting variables that were either significant in univariable analysis or had policy-relevant implications. We fit logistic regression models using backward selection to arrive at final models with significant terms for each period. We used the Firth bias reduction method as necessary. All analyses were conducted using R, version 4.2.2 statistical software (R Foundation for Statistical Computing). Because community COVID-19 case rates were similar in all included districts and the primary outcome of SAR is less sensitive to community transmission rates than the outcome of total case count among students and staff, we did not include weekly community rates in the final models. Data from the Centers for Disease Control and Prevention’s Social Vulnerability Index (SVI)^12,13^ were used to assess the association between district-level SAR and SVI. The SVI is a validated measure that combines 16 vulnerability-associated factors in 4 domains. The SVI has been associated with COVID-19 incidence and mortality, and school poverty level has been associated with access to mitigation measures in schools.^14,15,16^ We separately analyzed the 4 SVI components for each district (socioeconomic status, household characteristics [age, single parenting, disability, and English language proficiency], minority status [race and ethnicity], and housing type and transportation) as well as the overall SVI (a composite measure of all 4 components). The SVI values were categorized into quartiles for the analysis, with higher quartiles indicating a higher SVI. The overall SVI for the included districts ranged between the 10th and 70th percentile; the districts with the greatest vulnerability in the state did not participate in the study.

## Results

For F20/S21, 8 K-12 public school districts (70 schools with >33 000 enrolled students) participated in the contact tracing study (Table 1). During F20/S21, there were 435 index cases (151 staff, 216 students, and 68 missing role) with 1771 school-based contacts (278 staff, 1492 students, and 1 missing role). Of the 1771 contacts, 1327 (74.9%) underwent testing, 39 of these 1327 (2.9%) contacts tested positive for SARS-CoV-2. Of the 39 positive contacts, 10 (25.6%) had clear out-of-school exposures and were deemed not school-associated transmissions and excluded from the base-case SAR calculations. Twenty-nine contacts (74.4%) were deemed possible or probable school-associated transmissions, resulting in a school-associated SAR of 2.2% (lower bound, 1.6%; upper bound, 26.7%). The SAR ranged by district from 0.0% (lower bound, 0.0%; upper bound, 0.0%) to 11.9% (lower bound, 11.0%; upper bound, 19.2%). In a sensitivity analysis in which all contacts who tested positive for SARS-CoV-2 (including those deemed not school-associated transmissions) were included in the F20/S21 SAR calculation, the SAR was 2.9% (lower bound, 2.2%; upper bound 27.1%).

**Table 1.** SARS-CoV-2 Cases, Contacts, and Secondary Transmission by Period and District

Period and district	No. of students, faculty, and staff						SAR		
	Index cases	Contacts	Contacts tested	In-school transmissions					
				Possible	Probable	Total	%	Lower bound,^a^ %	Upper bound,^b^ %
Fall 2020/spring 21									
A	71	416	233	0	2	2	0.9	0.5	44.5
B	10	73	67	5	3	8	11.9	11.0	19.2
C	20	89	89	1	0	1	1.1	1.1	1.1
D	117	699	582	3	3	6	1.0	0.9	17.6
E	20	9	9	0	0	0	0.0	0.0	0.0
F	165	358	223	3	6	9	4.0	2.5	40.2
G	27	27	25	2	0	2	8.0	7.4	14.8
H	5	100	99	1	0	1	1.0	1.0	2.0
Total	435	1771	1327	15	14	29	2.2	1.6	26.7
Fall 2021									
A	38	408	342	0	4	4	1.2	1.0	17.2
C	70	498	498	16	0	16	3.2	3.2	3.2
G	99	178	175	3	11	14	8.0	7.9	9.6
I	92	529	520	6	4	10	1.9	1.9	3.6
School A	10	60	59	0	0	0	0.0	0.0	1.7
Total	309	1673	1594	25	19	44	2.8	2.6	7.4
Overall	744	3444	2921	40	33	73	2.5	2.1	17.3

Abbreviation: SAR, secondary attack rate (calculated as [probable and possible transmissions / contacts tested] × 100).

^a^ Assuming no untested contacts acquired infection.

^b^ Assuming all untested contacts acquired infection.

For F21, 4 K-12 public school districts and 1 prekindergarten through grade 9 private school (34 schools with >18 000 enrolled students) participated (Table 1). During F21, there were 309 index cases (37 staff, 207 students, and 65 missing role) with 1673 school-based contacts (107 staff and 1566 students). Of the 1673 contacts, 1594 (95.3%) underwent testing and 46 of the 1594 (2.8%) tested positive for SARS-CoV-2. Of the 46 positive contacts, 2 had clear out-of-school exposures and were excluded from the base-case SAR calculations. Forty-four were deemed possible or probable school-associated transmissions, resulting in a school-associated SAR of 2.8% (lower bound, 2.6%; upper bound, 7.4%). The SAR ranged by district from 0.0% (lower bound, 0.0%; upper bound, 1.7%) to 8.0% (lower bound, 7.9%; upper bound, 9.6%). In a sensitivity analysis in which all contacts who tested positive (including those deemed not school-associated transmissions) were included in the F21 SAR calculation, the SAR was 2.9% (lower bound, 2.7%; upper bound, 7.5%).

Tables 2 and 3 include the number of school-associated transmissions and SAR calculations according to different exposure types for F20/S21 and F21, respectively. During F20/S21, the unadjusted SAR was significantly higher if the exposure occurred at lunch, if both the index case and contact were unmasked, and if the index case had been tested because of an in-school exposure (Table 2). Higher overall SVI quartile (indicating greater social vulnerability) and higher socioeconomic SVI quartile were also associated with a higher SAR in univariable analysis (Table 2).

**Table 2.** Secondary Transmission and Exposure Characteristics, Fall 2020/Spring 2021 (Univariable Analysis)

Exposure	No. of students, faculty, and staff^a^					SAR, %	Fisher exact *P* value
	Index cases	Contacts	Contacts tested	No in-school transmission	In-school transmission		
Contact role							
Staff	151	278	233	224	9	3.9	.08
Student	216	1492	1093	1073	20	1.8	
Contact grade level and staff							
Prekindergarten, kindergarten, elementary^b^	119	793	628	617	11	1.8	.18
Middle school^c^	40	205	136	133	3	2.2	
High school^d^	36	328	255	251	4	1.6	
Staff	151	278	233	224	9	3.9	
Classroom exposure							
No	114	480	368	356	12	3.3	.14
Yes	187	1291	959	942	17	1.8	
Lunch exposure							
No	258	1728	1291	1266	25	1.9	.007
Yes	18	43	36	32	4	11.1	
Sports exposure							
No	254	1532	1152	1128	24	2.1	.58
Yes	19	239	175	170	5	2.9	
Distance during exposure, ft							
<3	20	82	81	77	4	4.9	.13
<6 (Not specified)	210	1248	927	910	17	1.8	
3-6	48	431	311	303	8	2.6	
Masking during exposure							
Both case and contact masked	250	1687	1256	1235	21	1.7	<.001
Case masked and contact unmasked	3	3	2	1	1	50.0	
Neither masked	28	67	60	53	7	11.7	
Semester							
Fall 2020	95	541	384	377	7	1.8	.68
Spring 2021	171	1211	938	916	22	2.3	
Index case role							
Staff	94	495	360	347	13	3.6	.04
Student	173	1276	967	951	16	1.7	
Index case grade level and staff							
Prekindergarten, kindergarten, elementary^b^	81	621	504	498	6	1.2	.12
Middle school^c^	39	224	145	142	3	2.1	
High school^d^	51	407	295	288	7	2.4	
Staff	94	495	360	347	13	3.6	
Means of identifying index case							
In-school close contact	5	13	11	9	2	18.2	.04
Out-of-school contact	83	588	413	408	5	1.2	
School-based asymptomatic testing program	57	384	325	318	7	2.2	
Tested because of symptoms	120	766	569	554	15	2.6	
Tested before or after travel	2	20	9	9	0	0	
Index case symptoms							
No	112	741	555	547	8	1.4	.13
Yes	155	1030	772	751	21	2.7	
SVI overall quartile^e^							
<0.25 (Lowest)	91	797	680	673	7	1.0	<.001
0.25-0.50	84	543	357	352	5	1.4	
0.51-0.75 (Highest)	92	431	290	273	17	5.9	
SVI SES quartile^e^							
<0.25 (Lowest)	162	1304	1003	993	10	1.0	<.001
0.25-0.50	95	394	257	246	11	4.3	
0.51-0.75 (Highest)	10	73	67	59	8	11.9	

Abbreviations: SAR, secondary attack rate (calculated as [probable and possible transmissions / contacts tested] × 100); SES, socioeconomic status; SVI, Social Vulnerability Index.

^a^ Unknown or missing values were removed if the number of unknown or missing values comprised <5% of the total number of contacts tested for each exposure category.

^b^ Grades 1 through 5.

^c^ Grades 6 through 8.

^d^ Grades 9 through 12.

^e^ Overall quartiles as found in the school districts included in the study.

**Table 3.** Secondary Transmission and Exposure Characteristics, Fall 2021 (Univariable Analysis)

Exposure	No. of students, faculty, and staff^a^					SAR, %	Fisher exact *P* value
	Index cases	Contacts	Contacts tested	No in-school transmission	In-school transmission		
Contact role							
Staff	37	107	107	107	0	0	.07
Student	207	1566	1487	1443	44	3.0	
Contact grade level and staff							
Prekindergarten, kindergarten, and elementary^b^	143	940	888	847	41	4.6	<.001
Middle school^c^	44	381	374	372	2	0.5	
High school^d^	24	237	220	219	1	0.5	
Staff	37	107	107	107	0	0	
Not available	4	8	5	5	0	0	
Classroom exposure							
No	132	497	485	479	6	1.2	.01
Yes	187	1176	1109	1071	38	3.4	
Lunch exposure							
No	207	1294	1218	1180	38	3.1	.15
Yes	106	379	376	370	6	1.6	
Sports exposure							
No	214	1658	1580	1536	44	2.8	>.99
Yes	2	15	14	14	0	0	
Distance during exposure, ft							
<3	168	1214	1203	1174	29	2.4	.003
<6 (Not specified)	13	72	68	68	0	0	
3-6	35	177	116	115	1	0.9	
Unknown	37	210	207	193	14	6.8	
Masking during exposure							
Both case and contact masked	172	1042	970	948	22	2.3	.003
Case masked and contact unmasked	4	8	8	8	0	0	
Neither masked	109	411	407	400	7	1.7	
Unknown	33	212	209	194	15	7.2	
Quarantine/testing approach							
TTS	186	1110	1110	1079	31	2.8	<.001
Quarantine	36	73	64	58	6	9.4	
Vaccinated; tested on day 3-5	55	350	350	350	0	0	
Other	19	77	10	9	1	10.0	
Contact vaccination status							
Fully vaccinated	65	410	398	398	0	0	<.001
Partially vaccinated	22	66	57	54	3	5.3	
Unvaccinated	194	1183	1128	1087	41	3.6	
Index case role							
Staff	21	200	162	161	1	0.6	.12
Student	194	1473	1432	1389	43	3.0	
Index case grade level and staff							
Prekindergarten, kindergarten, and elementary^b^	131	869	843	804	39	4.6	<.001
Middle school^c^	42	388	379	376	3	0.8	
High school^d^	21	216	210	209	1	0.5	
Staff	21	200	162	161	1	0.6	
Means of identifying index case							
In-school close contact, enrolled in TTS program	7	52	52	51	1	1.9	.55
In-school close contact, not enrolled in TTS program	1	2	2	2	0	0	
Out-of-school contact	51	375	373	361	12	3.2	
School-based asymptomatic testing program	46	265	261	251	10	3.8	
Tested because of symptoms	109	969	896	875	21	2.3	
Index case symptoms							
No	74	526	520	501	19	3.7	.14
Yes	141	1147	1074	1049	25	2.3	
Index case vaccination status							
Fully vaccinated	32	347	319	318	1	0.3	.001
Partially vaccinated	6	52	51	51	0	0	
Unvaccinated	176	1263	1213	1170	43	3.5	
SVI overall quartile^e^							
<0.25 (Lowest)	67	498	498	482	16	3.2	.63
0.25-0.5 (Highest)	138	1115	1037	1009	28	2.7	
SVI SES quartile^e^							
<0.25 (Lowest)	173	1435	1360	1330	30	2.2	<.001
0.25-0.5 (Highest)	32	178	175	161	14	8.0	

Abbreviations: SAR, secondary attack rate (calculated as [probable and possible transmissions / contacts tested] × 100); SES, socioeconomic status; SVI, Social Vulnerability Index; TTS, test to stay.

^a^ Unknown or missing values were removed if the number of unknown or missing values comprised less than 5% of the total number of contacts tested for each exposure category.

^b^ Grades 1 through 5.

^c^ Grades 6 through 8.

^d^ Grades 9 through 12.

^e^ Overall quartiles as found in the school districts included in the study.

During F21, the unadjusted SAR was significantly higher if the contact or the index case was an elementary student (compared with older grade levels and staff), if the exposure occurred in the classroom, if the contact did not participate in the TTS program (eg, chose quarantine), if the contact was partially vaccinated or unvaccinated, and if the index-case individual was unvaccinated (Table 3). Higher socioeconomic SVI quartile was also associated with a higher SAR (Table 3).

More information about index cases, including the proportion of index cases with any school-associated transmission, is outlined in eTables 3 and 4 in Supplement 3. In univariable analysis, a higher district-level SVI quartile was associated with a higher proportion of index-cases who transmitted infection in the first period (F20/S21). During the second period (F21), a greater proportion of elementary student index cases (compared with those in other grade levels or staff) and unvaccinated index cases (compared with vaccinated and partially vaccinated index cases) transmitted infection. A greater proportion of index cases in districts with a higher socioeconomic SVI quartile transmitted infection during F21.

In the final fitted multivariable models, during F20/S21, mask use of both the index case and contact was associated with lower odds of school-associated transmission relative to mask nonuse (Table 4; Figure, A). With both individuals masked, there was an 88% decrease in relative odds of in-school transmission (odds ratio [OR], 0.12; 95% CI, 0.04-0.40) and an absolute risk decrease of 9.6% (95% CI, −1.9% to −29.0%). During F21, classroom exposure and vaccination of the contact were associated with transmission (Table 4; Figure, B). A fully vaccinated contact had a 96% decrease in relative odds of in-school transmission (OR, 0.04; 95% CI, 0.0-0.62) and an absolute risk decrease of 3.6% (95% CI, −2.7% to −4.6%). A classroom exposure carried more than twice the odds of in-school transmission (OR, 2.47; 95% CI, 1.07-5.66) and an absolute risk increase of 2.1% (95% CI, 0.6%-3.8%) compared with an out-of-classroom exposure. For both F20/S21 and F21, higher SVI score (overall and socioeconomic status component) was associated with greater odds of school-associated transmission (Table 4).

**Table 4.** Relative Odds of School-Associated Transmission, Fall 2020/Spring 2021 and Fall 2021 (Logistic Regression)

Exposure	OR (95% CI)	*P* value	Risk difference (95% CI)
**Fall 2020 and spring 2021**			
Class exposure (vs out-of-class exposure)	2.73 (0.97 to 8.66)	.07	0.019 (−0.003 to 0.044)
SVI SES quartile increase (vs lowest quartile)	4.32 (2.48 to 7.61)	<.001	0.046 (0.022 to 0.078)
Both masked (vs both unmasked)	0.12 (0.04 to 0.40)	<.001	−0.096 (−0.290 to −0.019)
**Fall 2021**			
Class exposure (vs out-of-class exposure)	2.47 (1.07 to 5.66)	.02	0.021 (0.006 to 0.038)
SVI SES quartile increase (vs lowest quartile)	2.91 (1.53 to 5.55)	.003	0.040 (0.012 to 0.071)
Fully vaccinated contact (vs unvaccinated)	0.04 (0.00 to 0.62)	<.001	−0.036 (−0.046 to −0.027)
Partially vaccinated contact (vs unvaccinated)	2.48 (0.79 to 7.75)	.16	0.039 (−0.037 to 0.110)

Abbreviations: OR, odds ratio; SES, socioeconomic status; SVI, Social Vulnerability Index.

**Figure.**
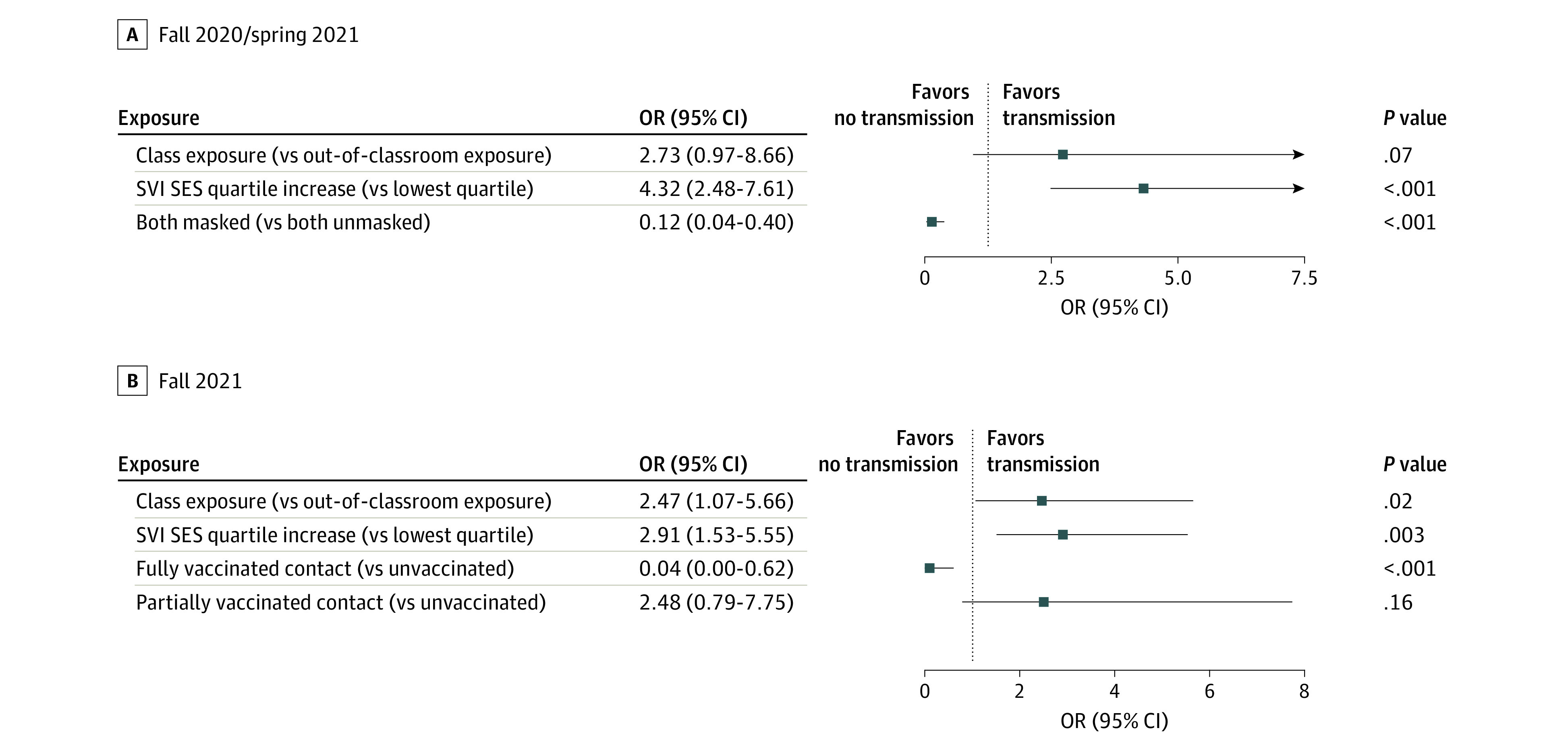
Factors Associated With the Odds of In-School Transmission for Fall 2020/Spring 2021 and Fall 2021 The C statistic for the fall 2020/spring 2021 model is 0.80 and for the fall 2021 model, 0.74. OR indicates odds ratio; SES, socioeconomic status; SVI, Social Vulnerability Index.

## Discussion

In this cohort study, based on detailed school-based contact tracing spanning 2 academic years and amid substantial changes in circulating SARS-CoV-2 variants and vaccine availability and uptake, the SAR among school-based contacts in Massachusetts was low. Our study expands the available literature on SARS-CoV-2 transmission in schools by providing an in-depth analysis of transmission context over time. During the first period (F20/S21), most schools were open and using a hybrid format, with reduced classroom density and greater distances between students in class. The original and Alpha variants were circulating, and vaccination was only available at the end of the academic year for staff and older students. The second period (F21) was characterized by the absence of a remote learning option, leading to greater classroom and lunchroom density, availability of vaccines for middle and high school students and staff, and circulation of the more transmissible Delta variant. Masking was required in classrooms during both periods; during F21, more nonclassroom school activities, such as sports, were unmasked. In both periods, available vaccines had high effectiveness in preventing transmission of circulating variants (original, Alpha, and Delta); both periods were before the widespread circulation of the Omicron variant. Despite important differences in factors associated with transmission, the SAR was low during both periods and similar to what has been reported in other studies.^1,2,3,4^

In this study, students and staff who lived in districts with greater social vulnerability, as measured by SVI, had a higher likelihood of infection through school-based exposure. It has long been recognized that the pandemic disproportionately affected communities with high social vulnerability.^17,18,19^ However, this study is the first to our knowledge to show that students and staff exposed to SARS-CoV-2 at school were more likely to become infected if they lived in districts with greater social vulnerability, even when vaccination status, distance, and other factors of transmission risk were considered. Because the outcome was SAR, this finding was independent of community rates of disease, and it held true during both study periods for reasons that are uncertain. Schools with more resources may have had more ability to implement ventilatory improvements, a factor known to reduce transmission in schools.^16^ Classroom density may be higher in lower-income neighborhoods.^20^ As we consider both the efficient use of limited current resources and the critical need for additional resources to improve student and staff health going forward, ensuring that resources are directed to districts in which transmission risk is higher may help to reduce both health and educational disparities.

Studies have shown that schools with mask policies had fewer cases of SARS-CoV-2 during the 2020-2021 school year,^8,21^ before widespread student and staff vaccination. While mask policies have been associated with reduced cases in schools, detailed contextual information about individual mask use has not previously been available. In this study, masking of both the index case and contact was protective against school-associated transmission during the 2020-2021 school year. Interestingly, in F21, despite a more transmissible variant (Delta) and greater classroom density, mask use was no longer found to be associated with reduced transmission; instead, vaccination of the in-school contact was the most protective factor. This finding suggests that at times of both high vaccination uptake and high vaccine effectiveness against the circulating variant, masking may be less preventive of transmission than vaccination. However, in settings with lower vaccination uptake, or when there is loss of vaccine effectiveness against circulating variants, masking may be more protective. Importantly, masking in schools may continue to be an important tool to prevent school-associated transmission when the effectiveness of vaccines against circulating variants and their effectiveness over time since vaccination are diminished.^22^

While distance between case and contact is known to be associated with transmission risk,^23^ in this study, the distance between the index case and contact was not found to be associated with transmission risk in schools. Importantly, the school-based teams conducting contact tracing were not always able to assess distance between cases and contacts. Distances were sometimes inferred based on policies in each location rather than on actual measurement of distance. Therefore, these data cannot be used to confirm that distance between case and contact is not relevant to transmission in schools.

During the 2020-2021 school year, we found no difference in transmission likelihood by age of the index case or contact as measured by students’ grade level. In F21, apparent differences in transmission by age may have been associated with age-related differences in vaccination. In the unadjusted analyses, elementary students were more likely to transmit infection and more likely to become infected when exposed than older students; however, in multivariable analysis, there was no association between age and transmission risk. This finding supports other data that suggest that transmission risk does not depend on age.^24,25,26,27^

In F21, the state of Massachusetts adopted a TTS program in which unvaccinated students and staff exposed to SARS-CoV-2 in schools were eligible to remain in school provided that they had a negative rapid antigen test result on all in-school days for 7 days following the exposure. Studies have shown that this approach is safe^28,29^ and associated with fewer lost learning days in the setting of exposure. In this study, unvaccinated students enrolled in the TTS program were less likely to acquire SARS-CoV-2 than unvaccinated students who were quarantined and tested. In addition, students who were identified as cases through the TTS program were not more likely to transmit infection than those identified as cases through other means (eg, symptomatic testing, out-of-school close contact). This finding suggests that exposed students who eventually tested positive but remained in school before their antigen test results became positive were not more likely than other students to transmit infection at school during that time, supporting the safety of TTS programs to minimize lost learning days.

### Limitations

This study had several important limitations. First, while the majority of school-based contacts were monitored for symptoms and underwent testing, not all contacts were tested for SARS-CoV-2. To address this limitation, we calculated the upper and lower bounds of SAR estimates, assuming that all untested contacts were truly infected or uninfected, respectively. These upper and lower bounds ranged widely, particularly for the first period (Table 1). Second, most contacts, unless enrolled in the TTS program, were tested only once. The sensitivity of a single test to confirm absence of transmission is imperfect.^30^ It is possible that the SAR would have been higher if exposed contacts were tested more frequently. Interestingly, contacts enrolled in the TTS program (who were tested more frequently but with rapid antigen tests) were not more likely to be diagnosed with SARS-CoV-2 than those not participating in TTS, but polymerase chain reaction testing was not performed. Third, contact tracing is imperfect, and some features that are potentially associated with transmission (eg, distance and masking during exposure) are subject to recall bias or unknown. Fourth, the likelihood of school-associated transmission for each contact who became infected was determined by school health staff; genomic sequencing of isolates to investigate the source of infection was not done. Fifth, the districts with the greatest vulnerability in the state were not included in the analysis; whether the correlations would hold if more these districts were included is unknown. Sixth, this study was conducted before the highly transmissible Omicron variant took hold. In January 2022, coincident with the arrival of the Omicron variant, contact tracing was no longer required in Massachusetts schools, and therefore we were not able to add a third study period to determine factors associated with school-based transmission during the Omicron era.

## Conclusions

The findings of this longitudinal cohort study of K-12 schools in Massachusetts, based on detailed school-based contact tracing during the 2020-2021 school year and fall semester of 2021, indicate that the SAR of SARS-CoV-2 among school-based contacts was low. The study highlights the importance of collecting data about school-based infectious disease incidence in order to identify factors associated with transmission, with the goal of acting on those that can be addressed through school-based or public health interventions. We provide an in-depth individual-level analysis of the context of transmission in schools, extending existing literature by highlighting the benefit of vaccination and masking to prevent transmission in school settings. The study also demonstrated that like the pandemic, factors associated with respiratory virus transmission risk in schools are not static and will be impacted by circulating variants, vaccination prevalence, vaccine effectiveness, testing protocols, and other factors. Importantly, this study highlights the importance of social vulnerability in transmission risk, suggesting that schools in districts of greater vulnerability must be provided with additional resources to optimize the health of students and staff. The generalizability of these data beyond the 2021-2022 school year remains uncertain. Ongoing surveillance of school-associated SARS-CoV-2 transmission in schools is critical to inform decisions about school-based mitigation measures as the pandemic continues to evolve.

## Notes

Supplement 1. **eTable 1.** K-12 Schools SARS-CoV-2 Contact Tracing Tracker, Fall 2020/Spring 2021

Supplement 2. **eTable 2.** K-12 Schools SARS-CoV-2 Contact Tracing Tracker, Fall 2021

Supplement 3. **eTable 3.** Index Case Characteristics and Number of Transmissions Per Index Case, Fall 2020/Spring 2021 (Univariable Analysis) **eTable 4. **Index Case Characteristics and Number of Transmissions Per Index Case, Fall 2021 (Univariable Analysis) **eFigure. **Vaccination Rates by Massachusetts District Over Time

Supplement 4.
Data Sharing Statement
