# Supplementary material for: Prevalence and Risk Factors for School-Associated Transmission of SARS-CoV-2
Source: JAMA Health Forum. 2023 Aug 4;4(8):e232310. doi: 10.1001/jamahealthforum.2023.2310 (PMC10403780; doi:10.1001/jamahealthforum.2023.2310)
Supplement: Supplement 3. — eTable 3. Index Case Characteristics and Number of Transmissions Per Index Case, Fall 2020/Spring 2021 (Univariable Analysis) eTable 4. Index Case Characteristics and Number of Transmissions Per Index Case, Fall 2021 (Univariable Analysis) eFigure. Vaccination Rates by Massachusetts District Over Time [file jamahealthforum-e232310-s003.pdf]

Supplemental Online Content

Nelson SB, Dugdale CM, Brenner IR, et al. Prevalence and risk factors for school-associated transmission of SARS-CoV-2. *JAMA Health Forum*. 2023;4(8):e232310. doi:10.1001/jamahealthforum.2023.2310

eTable 1. K-12 Schools SARS-CoV-2 Contact Tracing Tracker, Fall 2020/Spring 2021

eTable 2. K-12 Schools SARS-CoV-2 Contact Tracing Tracker, Fall 2021

eTable 3. Index Case Characteristics and Number of Transmissions Per Index Case, Fall 2020/Spring 2021 (Univariable Analysis)

eTable 4. Index Case Characteristics and Number of Transmissions Per Index Case, Fall 2021 (Univariable Analysis)

eFigure. Vaccination Rates by Massachusetts District Over Time

This supplemental material has been provided by the authors to give readers additional information about their work.

© 2023 Nelson SB et al. *JAMA Health Forum.*

eTable 1 (Supplement 1) and eTable 2 (Supplement 2): Data collection instruments

School staff were provided with these standardized data collection instruments. All data were collected prospectively during the study periods. School personnel responsible for data collection and data entry varied by district and were chosen by district teams; most were school nurses, nursing leaders, or administrators (e.g., Assistant Principals). In some cases, school nursing teams collected data on their own forms and translated data into study forms. In all cases, study investigators provided guidance at the beginning of the study period about the type of data needed, definitions of key terms, and how to use the data collection instrument. Study investigators were also available throughout the study to clarify these elements as needed. All cases and contacts were followed up (unless noted) during the study period; as this became infeasible during the Omicron surge, the study period concludes before widespread Omicron community transmission began in MA.

© 2023 Nelson SB et al. *JAMA Health Forum.*

eTable 3. Index Case Characteristics and Number of Transmissions Per Index Case, Fall 2020/Spring 2021 (Univariable Analysis)

Exposure	Index cases	Index cases with no contacts	Index cases with 1 or more contacts	Index cases with no transmissions[a]	Index cases with 1 or more transmissions[a]	Transmissions per index case[b]	Fisher's exact p value[c]
Index case role							0.507
Staff	149	55 (36.9%)	94 (63.1%)	84	10	0.14	
Student	286	113 (39.5%)	173 (60.5%)	159	14	0.09	
Index case grade category							0.65
PK/K/Elem	105	24 (22.9%)	81 (77.1%)	75	6	0.07	
MS	70	31 (44.3%)	39 (55.7%)	37	2	0.08	
HS	103	58 (56.3%)	45 (43.7%)	39	6	0.16	
Staff	149	55 (36.9%)	94 (63.1%)	84	10	0.14	
Unknown[d]	8	0 (0%)	8 (100%)	8	0	0	
District							<0.001
District A	71	0 (0%)	71 (100%)	69	2	0.03	
District B	10	0 (0%)	10 (100%)	3	7	0.8	
District C	20	4 (20%)	16 (80%)	15	1	0.06	
District D	117	47 (40.2%)	70 (59.8%)	65	5	0.09	
District E	20	15 (75%)	5 (25%)	5	0	0	
District F	165	83 (50.3%)	82 (49.7%)	75	7	0.11	
District G	27	19 (70.4%)	8 (29.6%)	7	1	0.25	
District H	5	0 (0%)	5 (100%)	4	1	0.2	
Semester							0.371
Fall 2020	155	61 (39.4%)	94 (60.6%)	88	6	0.07	
Spring 2021	277	107 (38.6%)	170 (61.4%)	152	18	0.13	

© 2023 Nelson SB et al. *JAMA Health Forum.*

Exposure	Index cases	Index cases with no contacts	Index cases with 1 or more contacts	Index cases with no transmissions[a]	Index cases with 1 or more transmissions[a]	Transmissions per index case[b]	Fisher's exact p value[c]
Means of identifying index case							0.32
In-school close contact	6	1 (16.7%)	5 (83.3%)	4	1	0.4	
Out of school close contact	128	45 (35.2%)	83 (64.8%)	79	4	0.06	
School-based asymptomatic testing	100	43 (43%)	57 (57%)	52	5	0.12	
Tested because of symptoms	194	74 (38.1%)	120 (61.9%)	106	14	0.12	
Tested before or after travel	5	3 (60%)	2 (40%)	2	0	0	
Unknown[d]	2	2 (100%)	0 (0%)	0	0	-	
Index case number of transmissions							<0.001
0	411	168 (40.9%)	243 (59.1%)	243	0	0	
1	19	0 (0%)	19 (100%)	0	19	1	
2	5	0 (0%)	5 (100%)	0	5	2	
SVI overall quartile							0.041
1	157	66 (42%)	91 (58%)	85	6	0.08	
2	103	19 (18.4%)	84 (81.6%)	80	4	0.06	
3	175	83 (47.4%)	92 (52.6%)	78	14	0.18	
SVI SES quartile							<0.001
1	213	51 (23.9%)	162 (76.1%)	153	9	0.06	
2	212	117 (55.2%)	95 (44.8%)	87	8	0.12	
3	10	0 (0%)	10 (100%)	3	7	0.8	

a. Of cases with at least one contact
b. Total number of transmissions divided by the number of index cases with 1 or more contacts
c. Comparing transmissions per index case between the rows in each section
d. All unknown or missing data are included in these supplemental tables.

Abbreviations: PK/K/Elem: Pre-Kindergarten, Kindergarten, and Elementary school (grades 1-5); MS: Middle school (grades 6-8); HS: High school (grades 9-12); SVI: Social Vulnerability Index; SES: Socioeconomic status; N/A: Not available.

© 2023 Nelson SB et al. *JAMA Health Forum.*

eTable 4. Index Case Characteristics and Number of Transmissions Per Index Case, Fall 2021 (Univariable Analysis)

Exposure	Index cases	Index cases with no contacts	Index cases with 1 or more contacts	Index cases with no transmissions[a]	Index cases with 1 or more transmissions[a]	Transmissions per index case[b]	Fisher's exact p value[c]
Index case role							0.322
Staff	28	7 (25%)	21 (75%)	20	1	0.05	
Student	281	87 (31%)	194 (69%)	165	29	0.22	
Index case grade category							0.072
PK/K/Elem	188	57 (30.3%)	131 (69.7%)	106	25	0.3	
MS	51	9 (17.6%)	42 (82.4%)	39	3	0.07	
HS	39	18 (46.2%)	21 (53.8%)	20	1	0.05	
Staff	28	7 (25%)	21 (75%)	20	1	0.05	
Unknown[d]	3	3 (100%)	0 (0%)	0	0	-	
District							0.297
District A	38	0 (0%)	38 (100%)	35	3	0.11	
District C	70	3 (4.3%)	67 (95.7%)	54	13	0.24	
District I	92	24 (26.1%)	68 (73.9%)	60	8	0.15	
School A	10	0 (0%)	10 (100%)	10	0	0	
District G	99	67 (67.7%)	32 (32.3%)	26	6	0.44	
Means of identifying index case							0.949
In-school close contact enrolled in TTS	14	7 (50%)	7 (50%)	6	1	0.14	
In-school close contact not enrolled in TTS	7	6 (85.7%)	1 (14.3%)	1	0	0	
Out-of-school contact	74	23 (31.1%)	51 (68.9%)	43	8	0.24	
School-based asymptomatic testing	80	34 (42.5%)	46 (57.5%)	39	7	0.22	
Tested because of symptoms	124	15 (12.1%)	109 (87.9%)	95	14	0.19	
Unknown[d]	10	9 (90%)	1 (10%)	1	0	0	
Index case vaccination status							0.152
Fully vaccinated	44	12 (27.3%)	32 (72.7%)	31	1	0.03	
Partially vaccinated	9	3 (33.3%)	6 (66.7%)	6	0	0	
Unvaccinated	245	69 (28.2%)	176 (71.8%)	147	29	0.24	
Unknown[d]	11	10 (90.9%)	1 (9.1%)	1	0	0	

© 2023 Nelson SB et al. *JAMA Health Forum.*

Exposure	Index cases	Index cases with no contacts	Index cases with 1 or more contacts	Index cases with no transmissions[a]	Index cases with 1 or more transmissions[a]	Transmissions per index case[b]	Fisher's exact p value[c]
Index case number of transmissions							<0.001
0	279	94 (33.7%)	185 (66.3%)	185	0	0	
1	21	0 (0%)	21 (100%)	0	21	1	
2	6	0 (0%)	6 (100%)	0	6	2	
3	1	0 (0%)	1 (100%)	0	1	3	
4	2	0 (0%)	2 (100%)	0	2	4	
SVI overall quartile							0.208
1	70	3 (4.3%)	67 (95.7%)	54	13	0.24	
2	229	91 (39.7%)	138 (60.3%)	121	17	0.2	
SVI SES quartile							0.428
1	200	27 (13.5%)	173 (86.5%)	149	24	0.17	
2	99	67 (67.7%)	32 (32.3%)	26	6	0.44	

a. Of cases with at least one contact
b. Total number of transmissions divided by the number of index cases with 1 or more contacts
c. Comparing transmissions per index case between the rows in each section
d. All unknown or missing data are included in these supplemental tables.

Abbreviations: PK/K/Elem: Pre-Kindergarten, Kindergarten, and Elementary school (grades 1-5); MS: Middle school (grades 6-8); HS: High school (grades 9-12); SVI: Social Vulnerability Index; SES: Socioeconomic status; TTS: Test-to-Stay program; N/A: Not available

© 2023 Nelson SB et al. *JAMA Health Forum.*

eFigure. Vaccination Rates by Massachusetts District Over Time

March through May 2021 (when vaccines first became available):

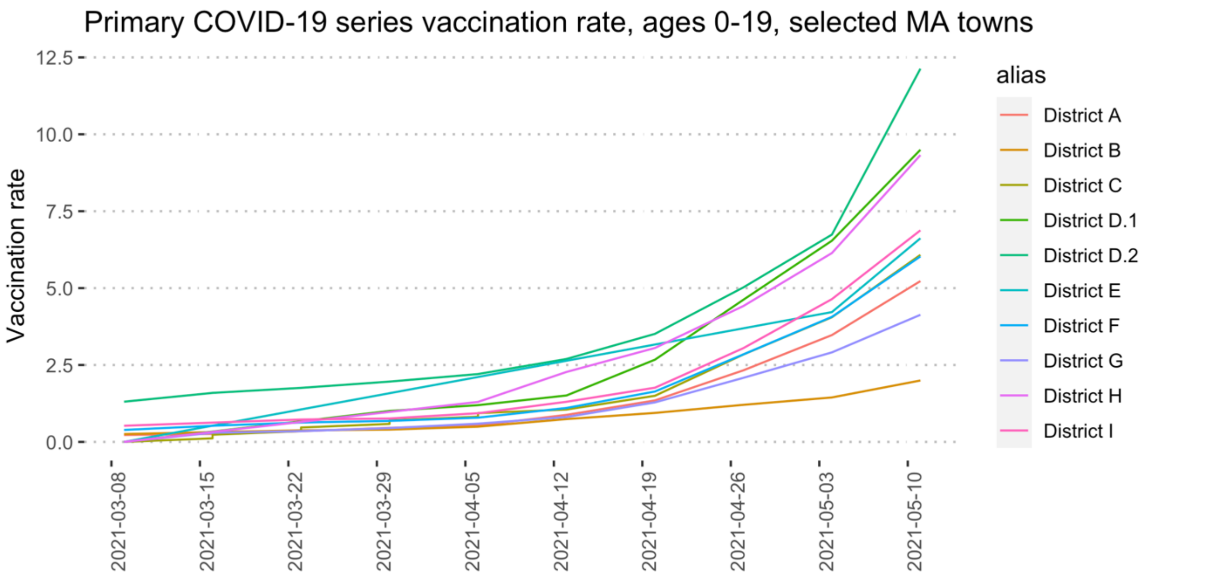

Once those 12+ became eligible, MA reported rates separately by age group (May 2021-April 2022):

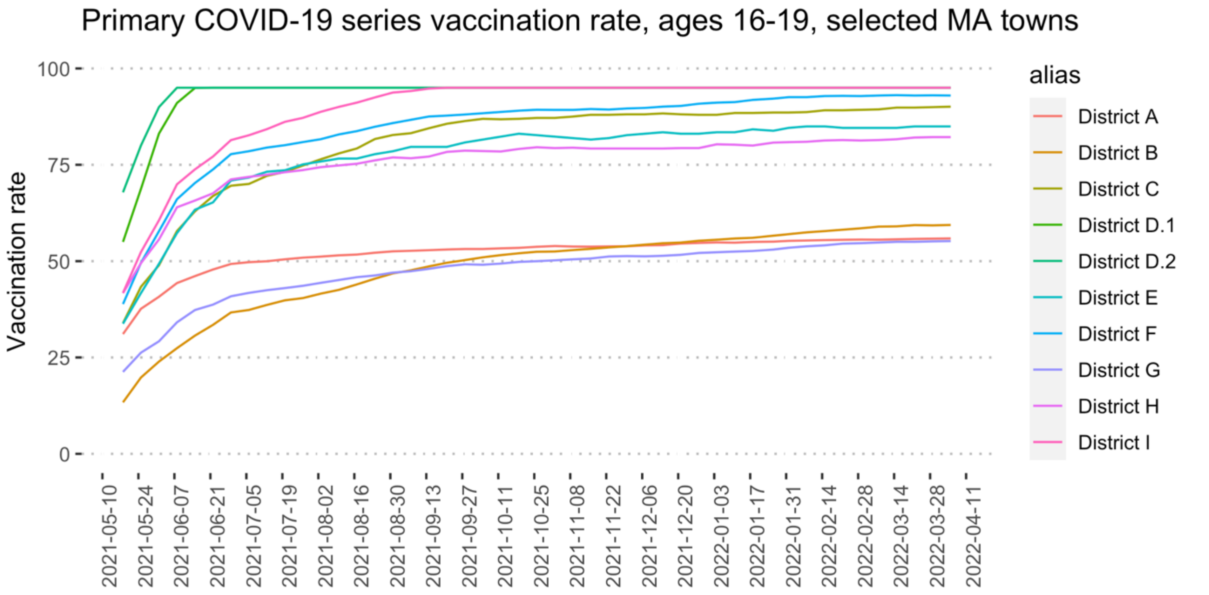

© 2023 Nelson SB et al. *JAMA Health Forum.*

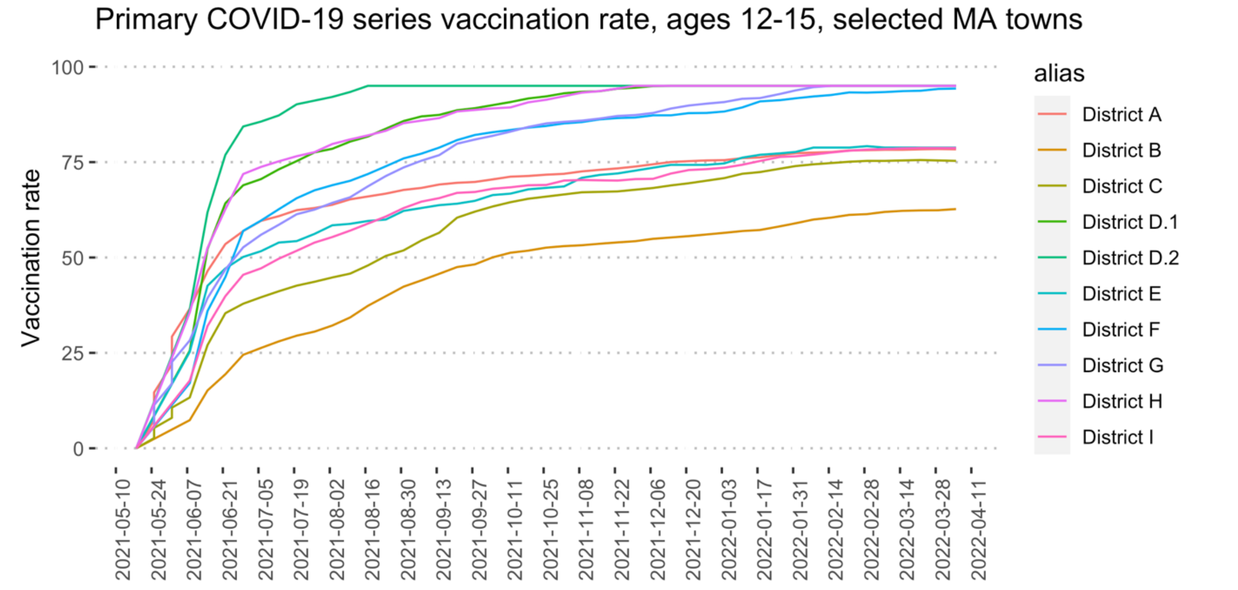

Ages 5-11 became eligible for vaccination in November 2021 (November 2021 through April 2022)

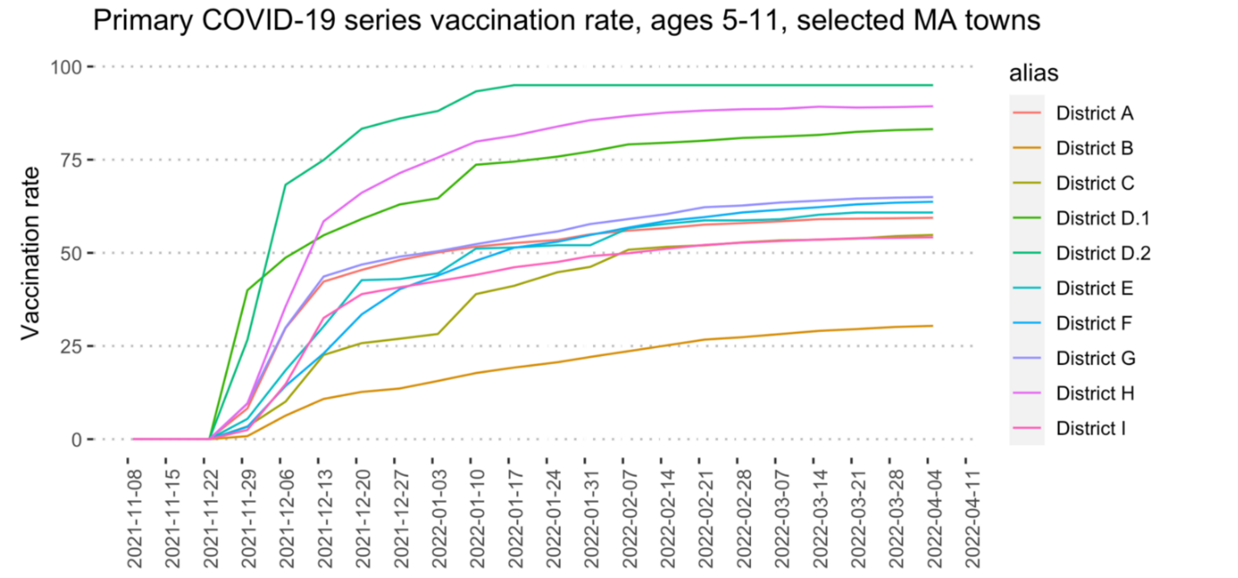

District D includes 2 towns; vaccination rates were reportedly separately for these towns.

© 2023 Nelson SB et al. *JAMA Health Forum.*
