# Supplementary material for: Prevalence and Risk Factors for School-Associated Transmission of SARS-CoV-2
Source: JAMA Health Forum. 2023 Aug 4;4(8):e232310. doi: 10.1001/jamahealthforum.2023.2310 (PMC10403780; doi:10.1001/jamahealthforum.2023.2310)
Supplement: Supplement 4. — Data Sharing Statement [file jamahealthforum-e232310-s004.pdf]

Data Sharing Statement

Nelson. Prevalence and Risk Factors for School-Associated Transmission of SARS-CoV-2. *JAMA Health Forum*. Published August 04, 2023. doi:10.1001/jamahealthforum.2023.2310

Data
Data available: Yes
Data types: Deidentified participant data
How to access data: Data from individual districts will be shared on reasonable request. Requests should be addressed to Dr. Andrea Ciaranello (aciaranello@mgh.harvard.edu)
When available: With publication

Supporting Documents
Document types: None

Additional Information
Who can access the data: Data will be made available to interested users upon successful review of data request as well as completion of any relevant data use agreements.
Types of analyses: Requests should articulate intended purpose.
Mechanisms of data availability: Data from individual districts may require data use agreements.
